# A Quantitative Exploration of Australian Dog Breeders’ Breeding Goals, Puppy Rearing Practices and Approaches to Socialisation

**DOI:** 10.3390/ani15152302

**Published:** 2025-08-06

**Authors:** Jessica K. Dawson, Deanna L. Tepper, Matthew B. Ruby, Tiffani J. Howell, Pauleen C. Bennett

**Affiliations:** 1 Anthrozoology Research Group, La Trobe University, Melbourne, VIC 3086, Australia; d.tepper@latrobe.edu.au (D.L.T.); t.howell@latrobe.edu.au (T.J.H.); pauleen.bennett@latrobe.edu.au (P.C.B.); 2 School of Psychology and Public Health, La Trobe University, Melbourne, VIC 3086, Australia; m.ruby@latrobe.edu.au

**Keywords:** breeder demographics, dog health and behaviour screening, dog temperament

## Abstract

Millions of puppies are welcomed into the homes of families around the world each year. However, understanding the ways in which puppies are bred and raised by their breeders, as well as the perspectives and perceptions underpinning these practices, is still in its infancy. The current study administered an online survey to 200 Australian dog breeders to investigate their breeding program characteristics, breeding dog selection, understanding of the importance of early experiences in puppyhood, and the extent and diversity of their puppy rearing and socialisation practices. Results indicated that breeders were motivated by breed improvement and producing dogs for themselves rather than providing companion dogs, despite most of their puppies being placed in companionship roles. The participating breeders also acknowledged the important role they play in shaping puppies’ behaviour and temperament, which was reflected in both their breeding dog selection and in their rearing and socialisation practices. The majority of breeders housed their litters within their residence for the initial weeks of life but the socialisation experiences they provided were variable in type and frequency. Longer-term breeders and those with larger, more intensive programs reported providing human-focused socialisation experiences less frequently, though the correlational nature of these findings require cautious interpretation. Whilst future research should endeavor to explore these results more comprehensively among a more diverse sample, these findings provide valuable insight into the breeding, rearing, and socialisation process undertaken by dog breeders in Australia.

## Simple Summary

Each year, thousands of puppies around the world join families, yet we still know little about how breeders raise them or what shapes their decisions. This study surveyed Australian dog breeders to learn more about their breeding program, how they choose their breeding dogs, what they know about early puppy development, and how they rear and socialize their litters. Most breeders were motivated by improving the breed, despite most puppies sold as pets. Breeders recognised that they have an important role in shaping their puppies’ future behaviour and made choices during breeding and rearing with this in mind. Longer-term breeders or those with larger programs appeared to socialize their puppies with new humans less frequently, although more research is needed to fully understand this. These findings offer insight into how breeders raise their litters and may be utilized to inform guidelines for the dog breeding industry, as well as in assisting breeders in making decisions that support puppy development and prepare them for life as family companions.

## 1. Introduction

Most companion dogs in higher-income nations, such as Australia, the United Kingdom, and the United States, are surgically altered to prevent breeding [1]. Hence, people usually acquire their dogs directly from breeders or through intermediaries like pet stores or rescue organizations [2]. As a result, dog breeding has become a large and profitable industry [3]. Each year an estimated 850,000 purebred puppies are sold in the United Kingdom [4], while Australian breeders supply around 500,000 puppies [5], and the American Kennel Club registered nearly 300,000 litters in 2023 [6]. These figures likely underestimate the size of the breeding industry because of varying registration requirements, existence of multiple registries, and demand for unregistered dogs. Breeders of purebred or ‘pedigree’ dogs typically register with different organizations than those who focus on crossbreeds, such as the poodle mixes that have grown exponentially in popularity in recent decades [7]. Breeders also exhibit considerable variation in scale, spanning from modest, occasional breeders who produce one or two litters annually to expansive commercial operations that generate hundreds of litters each year [8].

Regardless of type or scale, the role that breeders play in the development of dogs fit for contemporary lifestyles is important. Modern dog owners typically desire dogs who are healthy, friendly, affectionate, obedient, and safe with children [9,10]. Whilst ongoing training and healthcare throughout a dog’s life are integral to maximise these traits, the practices undertaken by the dog’s breeder also play a role. Selection of appropriate breeding dogs is important. Many canine health conditions are heritable and detectable through genetic tests [11]. Breeders can also consider factors, such as genetic diversity and the presence of morphological traits, like brachycephaly or dwarfism, that may impact the health and wellbeing of puppies they produce [12,13]. Heritability of temperament traits important for dogs living alongside humans as companions, such as sociability and trainability, has also been established [14,15].

The way that breeders raise their puppies is also impactful. Due to legislative requirements in several countries [16,17,18], puppies typically spend the first 8 weeks of their lives in the care of their breeder. This coincides with a crucial ‘socialisation period’, whereby between approximately 3 to 12–14 weeks of age puppies are most sensitive to both positive and negative external influences [19]. It is widely agreed that puppies should be appropriately socialized and desensitized to a wide range of experiences during this period [20]. However, despite the negative impact of insufficient socialisation experiences being well established [19,21], what constitutes optimal socialisation remains unknown [20].

Despite the roles breeders play in setting puppies up for success as adult companions, relatively little is known about the aims and motivations of dog breeders. Blackman et al. [22] sought to address this gap by investigating the aims and motivations of predominantly small-scale Australian dog breeders. They found that breeders perceived their breeding as a hobby and ranked their love for their breed as their primary motivation for commencing breeding. While financial gain was not initially a motivating factor, it became a significant factor over time. Additionally, most breeders participating in their study aimed to produce healthy companions that improved their breed, and prioritized the health, conformation, and temperament of breeding animals. It has not yet been empirically established whether specific breeding aims translate into differences in outcomes and practices, particularly rearing and socialisation practices.

Very few studies have examined how breeders select breeding dogs to meet their objectives. Czerwinski et al. [23] investigated factors Australian purebred dog breeders consider important when selecting breeding dogs, finding that breeders considered the quality of maternal care, offspring potential, and the temperament, genetics and overall health of their breeding females when making breeding decisions. However, the importance of some of these factors varied with how many breeds a breeder produced and the breed type or breed group their dogs belonged to. Only a small percentage of participating breeders utilized their own stud dogs. When selecting them, they considered conformation, size, pedigree, temperament, whether they complemented their female, and whether the sire produced healthy puppies previously. In Blackman et al. [22]’s study, a majority of breeders conducted health testing on their breeding stock and assumed responsibility for the short-term physical health of the puppies; however, only half considered themselves responsible for the mental health of their puppies in the short term. It is important to note that, in both existing studies, participants were predominantly small-scale pedigree dog breeders, referring to breeders of purebred dogs affiliated with their national kennel club. Larger-scale breeders and those involved in crossbreeding were not represented, despite their growing prevalence and popularity.

The field’s understanding of the rearing and socialisation practices undertaken by dog breeders is likewise still developing. Korbelik et al. [24] investigated early socialisation practices of Australian dog breeders and found that unregistered breeders reported having less experience in dog breeding and offered fewer socialisation opportunities than registered breeders. However, the participants in this study were all small-scale breeders and may not represent the experiences and practices undertaken by those who breed on a larger scale. A review by McMillan [25], which included studies from a number of countries, concluded that dogs obtained from larger-scale commercial breeders more frequently displayed behavioural issues in adulthood, such as increased fear and aggression towards humans and other dogs, than those obtained from other sources such as small-scale breeders. Though the author highlighted that these findings are possibly due in part to differences in the provision of socialisation experiences, an examination of what socialisation opportunities were provided to the dogs in each included study was not included. More recently, Dawson et al. [26] interviewed Australian dog breeders of varying program characteristics and found that breeders exposed their litters to some degree of socialisation when rearing them and provided a range of experiences that may be relevant to puppies destined for companionship roles, such as socialisation with a variety of humans and novel experiences and environments. There also appeared to be a variety of approaches to rearing and degrees of flexibility in their approaches, but the degree to which these findings can be generalised to the wider population of dog breeders is limited by the qualitative nature of the study, the small sample size, and the self-selected nature of the participants. Whilst previous research cumulatively shows that socialisation practices are implemented by breeders to some degree, what these practices are and how these vary across the spectrum of dog breeders is not yet clear.

The aim of this study was to investigate the perspectives, perceptions and practices of companion dog breeders. We focused specifically on Australian breeders, as the country’s regulatory framework is broadly comparable to international standards [27], and to facilitate comparison with prior research conducted in the same national context. Using a quantitative design, we explored general breeding program characteristics, such as breeding motivations, choice of breed, length of breeding, program size, and litter frequency. Additionally, we examined factors influencing the acquisition, evaluation, and selection of breeding dogs. To expand on previous research, we also investigated breeder perceptions of the importance of early experiences and the diversity and extent of socialization experiences they provide to their puppies. Finally, we explored whether specific socialisation experiences likely to be of particular importance for companion dogs, including exposure to a diverse range of humans, human apparel, and children under the age of 10, were associated with length of time breeding, litter frequency, and breeding program size.

## 2. Materials and Methods

### 2.1. Participants and Procedure

Individuals who were at least 18 years of age, were proficient in written English, identified as a dog breeder and had bred a litter within the previous 12 months were invited to participate in the study. Data collection occurred between June 2021 and April 2023. No modifications to the questionnaire or data analysis were conducted whilst the questionnaire remained open. Participants were recruited through dog breeding forums, and by sharing study information with members of various dog breeding organizations. This included branches of Dogs Australia. The questionnaire was also advertised on the Anthrozoology Research Group’s Facebook page using targeted advertising, whereby the researchers paid a small fee (AUD$50.00) for the advertisement to appear to people residing in Australia aged 18 years and over. Participants were encouraged to share the study with others who may be interested in participating.

### 2.2. Materials

A broad questionnaire was developed based on the qualitative findings of Dawson et al. [26] and input from the research team. The questionnaire was hosted on the online survey platform, REDCap [28], and contained 115 questions divided into five sections. Time to complete data were not collected for this study. However, prior to data collection, the survey was informally tested by members of the research team to estimate participant burden, with an approximate completion time of 30 min. This study is one component of a broader investigation into dog breeder perceptions and practices, which is still in progress. As such, only the questionnaire sections relevant to this publication are detailed here. The full questionnaire is provided in Supplementary S1.

Section 1 contained 26 questions concerning the participant’s breeding program. These included how long they had been breeding, their breeding goals, how many dogs they had in their program and the number of litters produced annually, as well as how they select breeding dogs.

Section 2 contained 47 questions concerning how participants raise their litters. It contained questions concerning their general whelping and rearing practices, such as whelping and rearing location(s), weaning age, and veterinary care provided during the rearing process. It also contained questions about the experiences provided and socialisation practices used during litter rearing, such as how important breeders consider them, which practices they typically use, and how often these occur. It included 19 socialisation experiences, such as “frequency of experiences with a diverse range of other humans (e.g., tall/short, elderly, with disability)”, that were presented on a 7-point Likert scale ranging from never to several times per day.

Section 3 contained 18 questions about breeders’ approaches to finding homes for their puppies, including the age at which puppies leave the breeder’s care and whether breeders maintain contact with new owners.

Section 4 contained 12 questions. These related to where participants obtain information about dog breeding and their beliefs about regulation within the breeding industry. The participants were also offered the opportunity to share any additional information they believed was not captured in the questionnaire through an open-ended text box.

Lastly, Section 5 contained 12 basic demographic questions, such as age, residence type and place, and living circumstances.

### 2.3. Analyses

All analyses were conducted using IBM SPSS Version 29. Descriptive statistics were utilized to summarize breeder and breeding program characteristics, and participant responses to questions pertaining to puppy socialisation experiences. The choice of descriptive statistic depended on the nature of the variable, with categorical and ordinal variables (e.g., Likert scale data) summarized using frequencies and percentages, and continuous variables (e.g., visual analog scale data) summarized using means, medians, and standard deviations. Ordinal variables related to breeding length, breeding program size, and litter frequencies were collapsed into groups based on response distributions after examining data quartiles in order to enhance interpretability. Spearman’s rho correlations were calculated to investigate relationships between socialisation experiences pertaining directly to human interactions (i.e., exposure to diverse humans, children under 10, and experiences of various human apparel such as umbrellas and hats) and the untransformed data for breeder length of time breeding, estimated litter frequency, and breeding program size. The litter frequency variable used was the estimated number of litters over the previous five years, as it showed greater variability than the one-year estimate. To reduce the risk of inflating the Type I error rate, no further correlations were performed. For Spearman’s rho, Prion and Haerling [29] report that ρ = 0 to 0.20 is negligible, 0.21 to 0.40 is weak, 0.41 to 0.60 is moderate, 0.61 to 0.80 is strong, and 0.81 to 1.00 is very strong. A threshold of *p* < 0.05 was used to determine statistical significance, with results achieving *p* < 0.01 and *p* < 0.001 noted where applicable.

## 3. Results

### 3.1. Participant Demographics

A total of 289 participants took part; 89 were removed for duplicated responses, not meeting the inclusion criteria, disingenuous data, or not completing the section of the questionnaire concerning puppy handling, experiences, and socialisation, as these questions were core to the aims of the study. A further 49 participants opened the questionnaire but did not answer any questions. Due to the anonymous nature of the study, it is not possible to determine whether any of these people completed the questionnaire on another occasion. Comprehensive demographic information for the remaining 200 participants can be found in Table 1. Participants were predominantly female with a mean age of 54.92 (*SD* = 12.86). Most participants resided in Victoria and lived in rural or suburban areas. Participants primarily had some form of post-secondary school education and reported their income as average or above average compared to others in their community.

### 3.2. Breeding Program Characteristics

The full results relating to participant breeding program characteristics are reported in Table 2. A vast majority of participants (*n* = 193, 96.50%) reported that they were a member of a canine club or organisation. Of those, most indicated they were members of Dogs Australia (*n* = 180, 90.00%), and over half (*n* = 118, 59.00%) were members of a breed type club or association (e.g., Gun Dog Club, Australian Labradoodle Association). A smaller portion (*n* = 11, 5.50%) were members of private breeding clubs or associations. It should be noted that participants could hold memberships in multiple organisations; thus, these categories are not mutually exclusive. When asked how long they had been breeding dogs, the greatest portion of participants indicated twenty years or longer, followed by six to fifteen years. Participants also predominantly reported that they bred purebred dogs and bred one breed of dog. When asked how many dogs they had in their breeding program, the median response was 3 males and/or females (*SD* = 3.60) and most owned three or fewer dogs at the time of participation. There were 102 breeds or breed mixes represented across the participants, with the most common breeds reportedly produced being Labrador Retrievers (*n* = 13, 5.46%), Golden Retrievers (*n* = 13, 5.46%), and Australian Shepherds (*n* = 9, 3.78%).

With regard to litter frequency, a majority of participants indicated that they usually breed one litter per year, with just over half estimating they had bred four or fewer litters in the past 5 years. Additionally, participants primarily reported that their breeding females typically produced 2 to 3 litters prior to retirement. Male dogs generally produced more litters, with over a quarter of participants reporting that their males typically produced 4–5 litters prior to retirement, and over 10% indicating that they utilise their males more than 10 times prior to retirement. For the puppies produced in their programs, the vast majority of participants indicated that they were most frequently homed as companion dogs.

### 3.3. Breeding Motivations and Breed Selection

Participants were asked about their general motivations for breeding dogs and were able to select as many options as applied to them and their program. The most popular reason was “to improve the breed(s) I have chosen”, followed by “to produce performance dogs for myself” and “for pleasure (i.e., a hobby)”. The least frequently reported reason was “for profit/financial gain”. When asked about their motivations for choosing a specific breed or breeds, the most frequently selected reasons were “behaviour/temperament traits” and “physical appearance/attractiveness”. Similar to their general breeding motivations, the least frequently endorsed reason for breed selection was “profitability”, closely followed by “public popularity”. A full summary of general breeding motivations and breed selection motivations can be seen in Table 3.

### 3.4. Breeding Dog Acquisition, Evaluation, and Selection

When looking to obtain a breeding dog, a majority of participants indicated that they would breed or raise it themselves (*n* = 182, 91.00%) or obtain it from friends or associates who were breeders (*n* = 138, 69.00%). A smaller number indicated they would consider obtaining from breeders they do not know (*n* = 27, 13.50%), such as through word of mouth or online advertisements. Several breeders utilised the ‘other’ textbox to indicate that they would only obtain breeding dogs from trusted sources, such as those that operated in similar circles, were registered with the same organisation as they were, or were what they deemed to be ‘ethical’ breeders by their standards.

Participants were also asked about their utilization of male dogs outside of their program for breeding purposes. Specifically, they were asked how often they used an outside male dog for breeding (0 = never, 100 = always), with the mean response being 61.42 (*Mdn* = 60.00, *SD* = 30.93). Additionally, they were asked how often they meet the outside male dog prior to incorporating it into their program (0 = never, 100 = always), and the mean response was 75.24 (*Mdn* = 90.00, *SD* = 29.79).

When asked about disorder and disease testing, labelled in the questionnaire as ‘health testing’, nearly all participants (*n* = 194, 97.00%) indicated they did at least some testing of their breeding dogs to evaluate their suitability for breeding. Most participants completed genetic profiling for heritable disorders, as well as hip, elbow, and eye screening. A full summary of reported testing rates can be seen in Table 4.

When evaluating dogs for their breeding program, almost all participants indicated that temperament of the dog was a factor they considered, whilst health testing results and the dog’s structural conformation were also frequently considered. On the other hand, the accessibility (e.g., geographical distance or the availability of fresh or frozen semen for breeding purposes) and performance status (e.g., achievement in conformation showing or sporting events) of the dog were considered important by fewer breeders. There were also several behavioural characteristics that most breeders considered would exclude dogs from their breeding program, with person-directed aggression being the most frequent, followed by fearfulness, anxiety, and dog-directed aggression. Behaviours such as inappropriate digging and a tendency to escape were less frequently reported as exclusionary characteristics. Many breeders also utilised an ‘other’ option in relevant questions to express their belief that many of the behavioural and temperament traits listed were learned and could be reduced with training. A full summary of breeding dog selection considerations and exclusionary traits can be seen in Table 5.

### 3.5. Breeder Perceptions of the Impact of Early Experiences on Development

When asked about the importance of the breeder’s role in shaping the adult behaviour and temperament of puppies (0 = not at all important, 100 = very important), the mean response was 92.12 (*Mdn* = 100, *SD* = 12.81). Similarly, participants rated the impact of experiences prior to leaving the breeder (*M* = 89.34, *Mdn* = 100, *SD* = 15.35) and experiences after leaving the breeder (*M* = 89.44, *Mdn* = 100, *SD* = 15.37) on a dog’s adult behaviour and temperament as both being very high.

### 3.6. Puppy Rearing Locations and Socialisation Experiences

With regard to where puppies were reared, most participants reported that puppies were inside their home for the neonatal (birth to two weeks of age; *n* = 181, 90.50%) and transitional (two to four weeks of age; *n* = 169, 84.50%) periods of development. Once they left the whelping area, participants reportedly kept their puppies primarily inside the home (*n* = 107, 53.50%), outside in a kennel setup that was often specifically designed for puppies (*n* = 38, 19.00%), or in their backyard area (*n* = 28, 14.00%). Nearly all participants reported that four or fewer people were involved in the day-to-day care of their litters (*n* = 191, 95.5%), with two people being most commonly reported (*n* = 90, 45.0%).

Participants predominantly indicated that they begin handling their puppies during the whelping process or immediately after (*n* = 190, 95.00%). After birth, the majority of participants indicated that their puppies experienced human handling or interaction several times per day when in the presence of their littermates (*n* = 195, 97.50%). Handling or interactions away from siblings were also most commonly conducted several times a day (*n* = 146, 73.00%), but once per day was also frequently reported (*n* = 38, 19.00%).

In recent years, several socialisation protocols have become available for those raising puppies, with some offered commercially and others freely accessible. Over half of the participants reported practicing one or more of these socialisation protocols when rearing their litters (*n* = 116, 58.00%). Of these, 71.55% (*n* = 83) practiced early neurological stimulation [30], 69.83% (*n* = 81) practiced Puppy Culture [31], and 5.17% (*n* = 6) utilized education programs produced by Avidog International [32]. Several breeders elected to use the ‘other’ textbox to clarify that, whilst they utilized information from the programs listed, they modified them to suit their breed or circumstances.

Participants were asked how frequently their puppies encounter various stimuli and experiences throughout the socialisation period (5 to 8 weeks of age; see Table 6). Exposures to household noises, outdoor surfaces, indoor surfaces, machinery, and sudden or loud noises were frequently reported to occur multiple times per day. Contrastingly, experiences with unfamiliar dogs, alone time away from littermates and familiar humans, and leashed walking were frequently reported to never occur. Exposure to diverse humans, such as those of different heights, and a variety of human apparel most frequently occurred several times per week, whilst exposure to children under the age of 10 years reportedly occurred most frequently once or twice during this period.

### 3.7. Correlations Between Human-Centered Socialisation Experiences and Breeding Program Characteristics

Spearman’s rho correlations between human-centered socialisation experiences between 5 and 8 weeks of life and length of time breeding, estimated litter frequency, and breeding program size can be seen in Table 7. Experiences with a diverse range of humans were weakly negatively correlated with breeding length, litter frequency, and breeding program size. Experiences with children under 10 years old were weakly negatively correlated with breeding length, and negligibly negatively correlated with breeding program size. Experiences with various human apparel were negligibly negatively correlated with breeding length and breeding program size, and weakly negatively correlated with litter frequency.

As breeding length, but not litter frequency or breeding program size, could be reasonably expected to be confounded by participant age, an additional Spearman’s rho correlation was conducted to investigate the relationship between participant age and breeding length. Results indicated a moderate positive correlation, ρ = 0.42, *p* < 0.001.

## 4. Discussion

The aim of this study was to improve understanding of the companion dog breeding industry by exploring the perspectives, perceptions and practices of companion dog breeders. We focused on Australian breeders due to their internationally comparable regulations [27] and to enable comparison with prior national studies. Participants included 200 dog breeders who collectively produced upwards of 100 different breeds or breed types. Over half of the participants reported having more than 15 years of experience in dog breeding. Nearly all were members of Dogs Australia and bred purebred dogs that were primarily homed as companions. Most focused on a single breed and operated on a small scale, typically with five or fewer dogs in their program, producing fewer than seven litters over a five-year period. This is in line with previously published data by Dogs Australia [5], who reported that the majority of breeders affiliated with their organisation produced fewer than four litters per year, with over 50% producing only a single litter annually.

The overwhelming representation of Dogs Australia members is unfortunate but not surprising, particularly given that previous studies have attracted similar samples [22,23]. Dogs Australia-affiliated breeders are believed to represent a small section of Australian dog breeders, with 27,042 memberships of Dogs Australia in 2024, only a portion of whom are involved in breeding [33]. According to Dogs Australia [5], members produce a marginal portion of puppies available for purchase by the general public each year. Whether this claim is accurate or not is difficult to ascertain. Most Australian states have enacted strict legislation governing dog breeding. In Victoria, for example, breeders are restricted to a maximum of fifty breeding females, and those wanting any more than ten must undergo an approval process involving their local and state governmental bodies [34]. Nonetheless, there is no central database of dog breeders, so it is impossible to determine the representativeness of the current sample.

The most frequently endorsed motivations for breeding centered around participants improving their chosen breed(s), producing their own performance dogs, or simply for pleasure or as a hobby, with very few breeders reporting being motivated by financial reasons. They also reportedly chose their breed(s) most commonly due to perceived behaviour, temperament, and physical traits, with few breeders taking public popularity or profitability of the breed into account. These findings are mostly in line with those of Blackman et al. [22] and are consistent with most participants being members of Dogs Australia, a body that emphasizes adherence to breed standards and encourages member engagement in dog-related activities as a means of legitimizing dogs as suitable for breeding [35]. However, contrary to the findings in our present study of financial gain not being an important consideration for the majority of participants, Blackman et al. [22] found financial gain to be a motivating factor for their Australian dog breeding participants, though not necessarily the primary factor.

This difference may reflect limitations in the phrasing of the questions in the current study or qualitative differences between the two samples, though the latter is difficult to assess due to limited participant information in the previous study. Regardless, the low importance placed on financial profitability in this study is interesting in terms of sustainability of the industry. It may reflect high costs associated with dog breeding or the commonly expressed motivation of participants’ breeding for pleasure or as a hobby, rather than to service a particular market need. Consistent with this, despite nearly 90% of breeders indicating their puppies are homed primarily as companions, just under 60% indicated producing companion dogs as a motivation for breeding, and less than half indicated that they took their breed’s suitability as pets into account when selecting them as their breed of choice. This suggests, at least in this sample, that there is a potential discrepancy between motivations for breeding and the eventual roles that most puppies fulfill. It would be interesting to explore this issue further in a sample more representative of breeders not affiliated with Dogs Australia, particularly those who do breed for companion homes and/or for profit. Future research should investigate whether breeder motivations impact the ways in which they rear puppies who are predominantly destined for companionship homes.

When making decisions around selecting breeding dogs, temperament was the most frequently reported consideration by breeders. Similar findings were reported by Czerwinski et al. [23] and Blackman et al. [22], who found that participants also considered the temperament of breeding dogs an important factor. The scope of the current study did not allow us to determine exactly what aspects of temperament were considered important, but it was of interest that, for most breeders’, person-directed aggression, fearfulness and anxiety would lead them to exclude dogs from their program. These findings are encouraging considering how highly dog owners value temperament [10,36], the genetic influences on temperament and behaviours, such as aggression and fearfulness [37], and the profound effects behavioural problems can have on dogs and owners [38,39,40]. However, many participants noted in the ‘other’ textbox that they believed the types of traits included in the list were due to training or management issues rather than a result of any hereditary disposition. Whilst a comprehensive understanding of the inherited elements of specific canine behaviours and temperament traits is still developing [41], it is generally accepted that most behavioural and temperament traits have a genetic component. At the same time, environmental factors like training and socialisation play a considerable role in their expression [42]. Selecting breeding dogs with desirable traits and excluding those with problematic behaviors is an important step towards producing dogs that suit modern family life [1,9].

The health of potential breeding dogs was also a consideration for most breeders, with an overwhelming majority indicating that they utilized disorder and disease testing when evaluating dogs for breeding purposes. This is again in line with Czerwinski et al. [23] and Blackman et al. [22], and is a promising result given both the importance that dog owners place on having a healthy companion [10,36], and a general increase in concern for the health of breeding dogs and the puppies they produce [13]. Breeders have been encouraged by researchers and are sometimes required by kennel clubs to conduct disorder and disease testing to improve health outcomes [43,44]. Whilst genetic screening was utilized by most participants, the utilization of other tests varied substantially and likely reflects variability in testing recommendations across different breeds. For example, registration of Labrador and Golden Retriever litters with Dogs Australia requires parent dogs to have hip and elbow scoring, whereas cardiac screening is particularly important for breeds like Cavalier King Charles Spaniels, Dobermanns and Boxers [45,46].

The majority of breeders acknowledged that they played an important role in shaping the behaviour and temperament of their puppies. They also acknowledged the importance of early experiences of puppies in their care. This encouraging finding is in line with previous research highlighting the considerable impact of decisions made during the breeding process and the experiences puppies are exposed to during their ‘critical period of socialisation’ [19]. Breeders also believed that experiences beyond the time spent with the breeder were impactful on adult behaviour and temperament, perhaps acknowledging limits to their influence once owners ‘take the wheel’ in shaping their puppy’s future behaviour through appropriate socialisation [47]. These results are somewhat contrary to those of Blackman et al. [22], who found that only half of their breeder sample indicated they felt some degree of responsibility for the short-term mental health of puppies they produce. This may indicate that breeder education programs are having a positive impact on breeder beliefs, although future research is required to explore the nuances in breeders’ beliefs about the roles both they and owners play in shaping adult temperament and behaviour.

Most breeders indicated they whelped and reared puppies until the end of the transitional period inside their home. As the puppies matured, just over half continued to keep them predominantly indoors, whilst others moved them into a backyard or a specialized kennel setup. The initial time spent inside a home is likely to be important developmentally, providing opportunities for socialisation and exposure to experiences common for dogs who live alongside human families. Majecka et al. [48] reported that, for puppies bred by small-scale Polish breeders, those reared inside the home were less likely to show aggressive or fearful behaviours and were more capable of coping with novel conditions when evaluated at seven to eight weeks of age than puppies reared in outdoor kennel setups with more limited human contact. These findings were attributed to potential environmental differences and frequency of exposure to humans, though this could not be causatively established. Whilst breeders in the current study predominantly indicated that their litters were first handled at birth and continued to be handled numerous times per day whilst in their care, nearly half indicated that their puppies moved outside of the home once large enough to leave the initial whelping area. It would be interesting to explore whether there is an optimal amount of time spent within the home for behavioural outcomes. Additionally, establishing whether the important factor is housing location, proximity to and interaction with humans, or a combination of both, would also be worthy of investigation.

Breeders demonstrated variability in the way they approached socializing their litters. Over half reported using published socialisation protocols, predominantly either Early Neurological Stimulation (ENS) [49] or the Puppy Culture program [31]. Both programs have rapidly grown in popularity in recent years, often being promoted as having scientific support. However, contemporary research does not illustrate any long-term benefits of ENS over and above normal handling [50,51]. Likewise, whilst some of the practices involved in the Puppy Culture program have a foundation in human and animal development literature, the efficacy of the program itself has not been evaluated empirically. It is possible that such programs provide a valuable framework or ‘road map’ for breeders to utilize when socializing their puppies, particularly for those with limited time and resources to dedicate to developing and implementing their own socialisation protocols. Future research should not only look at establishing the efficacy of such programs on dog outcomes, but also breeders’ motivations, experiences, and perceived outcomes when implementing them.

With regard to specific experiences breeders expose their litters to during the portion of the socialisation period in which puppies are typically still in their care (5 to 8 weeks) [19], the level of exposure varied across different types of experiences. Experiences that likely happen incidentally as a part of the rearing process, such as exposure to household or sudden noises, and exposure to indoor and outdoor surfaces, were the most likely to occur several times per day. Those that likely require more planning or independent administration, such as participating in a car journey, leashed walking, or alone time from humans and littermates, were the least frequent to occur. Likewise, experiences that may be perceived as risky to the health or safety of the puppy, such as exposure to animals of other species or unfamiliar dogs, were also less likely to occur or sometimes never occurred at all.

When looking specifically at experiences relating to human interactions, breeders typically exposed their litters to a diverse range of humans and human apparel, such as umbrellas and hats, several times per week. These findings are encouraging given the value many dog owners place on their dog being friendly and sociable beyond their immediate family [10,52], and the negative implications for dogs who exhibit fear or reactivity towards people, for not only the owner, but for the dog and the dog–owner relationship [53]. Exposure to children under ten happened less frequently, with most breeders indicating this occurred once or twice in this time. Though variation on this item was considerable, this trend is somewhat concerning. It is well established that people, particularly those with families, desire dogs who are safe around children [10,36,52]. Children also commonly have difficulty interpreting dog behaviour and are the most affected demographic in dog bite cases [54,55]. However, early exposure to children during the socialisation period can lead to more appropriate responses from dogs towards children [56]. Breeders should therefore be encouraged to expose puppies to children in a positive way early and often.

Lastly, we explored whether the frequency of experiences relating to human interactions were associated with various breeding program characteristics. Results indicated that having more litters over a five-year period and having more breeding dogs were associated with less frequent exposure of litters to diverse humans and human apparel. Having a larger breeding program was also associated with less frequent exposure of litters to children under ten, although the effect size for this relationship was statistically negligible. These findings suggest that those with larger, more intensive programs may be less inclined or less able to facilitate these experiences, but that caution should be exercised when interpreting these data. Given the potential difficulties larger-scale and commercial breeding programs may have in meeting the socialisation needs of their puppies [8,25], investigating this further, particularly in the Australian context, is warranted.

Length of breeding was associated with less frequent litter exposure to diverse humans, children under 10 years of age, and various human apparel. A subsequent analysis indicated that length of breeding was correlated with participant age, so it may be participant age, rather than how long they have been breeding for, which determines accessibility or willingness to expose puppies to these experiences. Future research should seek to disentangle this relationship, particularly given that a substantial portion of dog breeders may be both older adults and long-time breeders, as suggested by the mean age of participants and their reported length of time breeding in this study.

The findings of this study should be interpreted in light of several limitations. As mentioned previously, whilst efforts were made during recruitment to involve diverse breeder types, most participants were members of Dogs Australia, bred on a small scale and were relatively unconcerned with profitability or breeding dogs for companionship roles. As a result, it is unlikely that this study is representative of the broader population of Australian dog breeders. In particular, the findings are unlikely to reflect the experiences of larger-scale or commercial breeders, whose influence may be more substantial due to the volume of dogs they produce. Although large-scale dog breeding is actively discouraged in many Australian states, future research should focus heavily on recruiting breeders from a broader range of programs, with a particular focus on larger-scale breeders not registered with Dogs Australia and whose perspectives, perceptions and practices remain underrepresented in the literature but could inform a more comprehensive understanding of the industry. It may also be valuable to explore the influence of broader demographic factors, such as breeder age, income, and employment status, on breeding perceptions and practices. Likewise, while our focus on Australian breeders was purposeful, future research would also benefit from exploring breeder practices and motivations outside of Australia, as the findings of this study may not generalize to the wider population of dog breeders. Lastly, whilst the largely descriptive nature of this study provides valuable insights, future research with larger samples may further enhance the validity and reliability of these findings.

Even within the population of breeders associated with Dogs Australia, the study sample comprised respondents who chose to complete a comprehensive questionnaire about their program, suggesting a potential interest in best practices and confidence in their breeding approach. This may have led to socially desirable responding. Breeders who are members of Dogs Australia are bound by rules and regulations that may influence their reported practices when breeding and rearing dogs, as well as their reported breeding goals and motivations. For example, several participants noted in open-ended text boxes that they had an aversion to the word ‘pet’ when referring to their breeding purposes and practices and avoided response options when the word was included. This is possibly due to Dogs Australia’s code of ethics requiring breeders to breed to ‘improve the breed’ rather than to produce for the pet market exclusively [35].

It is also worth noting that the questions pertaining to socialisation practices likely did not capture the full range of activities that breeders may engage in when raising litters. Whilst the content of the questionnaire was informed by Dawson et al. [26]’s qualitative findings relating to socialisation practices, additional aspects of these practices remain unexplored. Likewise, the intentionality behind the practices investigated in this study, namely exposure to stimuli and experiences during the socialisation window, could not be established. These experiences may occur incidentally and, although still valuable, could be more susceptible to variation in response to changes in breeder circumstances than if they were intentionally engaged in. As a result, future research would benefit from investigating socialisation practices on a broader scale and establishing the intentionality of practices more conclusively.

## 5. Conclusions

Although dog breeders play a key role in producing the next generation of companion dogs, their breeding and rearing practices are not well understood. In this study, we found that most breeders reported being driven by a desire to improve their breed. While the majority stated that their puppies were placed in companion homes, many also indicated that producing for such homes was not their primary motivation. However, breeders did recognise their role in shaping puppies’ behaviour and temperament. This includes selecting breeding dogs based on temperament, but also broader rearing and socialisation practices. Most breeders reported housing their litters inside their residence for at least the first few weeks of life, with considerable variability in how they approached socialisation. Results also suggested that those who had been breeding for longer or had larger, more intensive programs may be less able or inclined to provide human-focused socialisation experiences. However, these findings should be interpreted tentatively, particularly considering the unrepresentative nature of the sample and the limited scope of the socialisation experiences investigated.

Collectively, the findings of this study contribute to the limited understanding of the perspectives and practices of dog breeders in Australia. The findings of this study are largely consistent with previous literature, confirming their replicability and contributing to a more robust evidence base for future research on dog breeding. Additionally, to the authors’ knowledge, the investigation of rearing and socialisation perceptions and practices is novel, particularly in the context of Australian dog breeders. Collectively, these findings extend the existing knowledge base on dog breeding practices and provide a foundation for future empirical investigations in this area. Further research, particularly involving a more diverse sample of breeders and a deeper examination of socialisation practices and their effects on later dog behaviour and temperament, would help build a more nuanced understanding of breeder practices and their implications. Such insights would be not only be valuable for enhancing outcomes for owners and dogs, but may also assist in developing evidence-based legislation and industry standards, helping to ensure that regulations guiding practices are most likely to maintain optimum welfare whilst guiding breeders in producing puppies best prepared for life as human companions.

## Figures and Tables

**Table 1:** Participant Demographics (*N* = 200).

	*n*	*%*
Gender		
Female	176	88.0
Male	21	10.5
Non-binary/third gender	1	0.5
Prefer not to say	1	0.5
State of Residence		
New South Wales	15	7.5
Queensland	11	5.5
South Australia	12	6.0
Victoria	142	71.0
Western Australia	9	4.5
Living Circumstances		
Urban (inner city)	6	3.0
Suburban (over 10 km/6 miles from the inner city)	55	27.5
Regional city (population (50,000 or more)	34	17.0
Country town/island (population less than 50,000)	38	19.0
Rural (not in a city or a town)	66	33.0
Highest Level of Education		
Year 10 or below (up to age 16 years)	9	4.5
Year 11 or 12 (above age 16 years)	18	9.0
Certificate, diploma, advanced diploma, associate degree, technical/trade qualification, TAFE	84	42.0
Undergraduate University (bachelor’s degree)	51	25.5
Postgraduate University (master’s degree/PhD)	38	19.0
Employment Status		
Employed full time (35 h or more per week)	69	34.5
Employed part time (up to 34 h per week)	54	27.0
Unemployed and not currently looking for work	3	1.5
Unemployed and currently looking for work	1	0.5
Engaged in home duties	6	3.0
Retired	60	30.0
Student	1	0.5
Unable to work	5	2.5
Income Relative to Others in Community		
Very below average	4	2.0
Below average	17	8.5
Average	112	56.0
Above Average	57	28.5
Very above average	8	4.0

Note. Discrepancies between the total sample size (*N* = 200) and the summed category counts/percentages reflect missing data for participants who did not answer specific questions.

**Table 2:** Participant Breeding Program Characteristics—Frequencies and Percentages.

	*n*	*%*
Breeding Length		
Less than five years	48	24.0
Six to fifteen years	49	24.5
Sixteen to nineteen years	12	6.0
Twenty years or longer	91	45.5
Breed Type		
Purebred dogs	189	94.5
Crossbred/designer bred dogs	9	4.5
Both purebred and crossbred/designer bred dogs	2	1.0
Number of Breeds		
One breed/breed mix	169	84.5
Two breeds/breed mixes	24	12.0
Three breeds/breed mixes	7	3.5
Breeding Program Size		
One dog or less	36	18.0
Two to three dogs	80	40.0
Four to five dogs	42	21.0
Six or more dogs	42	21.0
Litter Frequency per Year		
One litter	119	59.5
Two litters	48	24.0
Three or more litters	33	16.5
Litter Frequency Five Year Estimate		
One to two litters	49	22.5
Three to four litters	57	28.5
Five to seven litters	43	21.5
Eight or more litters	51	25.5
Total Number of Female Dog Litters Prior to Retirement		
One litter	12	6.0
Two to three litters	150	75.0
Four to five litters	38	19.0
Total Number of Male Dog Litters Prior to Retirement		
One litter	13	6.5
Two to three litters	44	22.0
Four to five litters	57	28.5
Six to ten litters	32	16.0
Ten to twenty litters	15	7.5
More than twenty litters	10	5.0
Primary Puppy Home Type		
Pet/companionship	177	88.5
Sporting (agility, obedience, etc.)	3	1.5
Working (assistance work, hunting, etc.)	5	2.5
Conformation showing	9	4.5

Note. Discrepancies between the total sample size (*N* = 200) and the summed category counts/percentages reflect missing data for participants who did not answer specific questions and/or instances where multiple response options were permitted to be selected.

**Table 3:** Frequencies and Percentages for Participant Breeding Motivations and Breed Selection Motivations.

	*n*	*%*
Dog Breeding Motivations		
To improve the breed(s) I have chosen	160	80.0
To produce performance dogs for myself (for breeding, showing, sporting, working, etc.)	144	72.0
For pleasure (i.e., a hobby)	142	71.0
To produce companion dogs for others (i.e., supply ‘pets’)	119	59.5
To produce performance dogs for others (for breeding, showing, sporting, working, etc.)	92	46.0
To produce companion dogs for yourself	76	38.0
For profit/financial gain	22	11.0
Reasons for Breed Selection		
Behaviour/temperament traits	172	86.0
Physical appearance/attractiveness	120	60.0
Suitability as pets	83	41.5
Breed history/origin	60	30.0
Ability to fulfill performance/working roles	57	28.5
Previous experience with the breed (e.g., had one as a child)	49	24.5
Tradition (e.g., parents/grandparents bred them)	10	5.0
Public popularity	5	2.5
Profitability	2	1.0

**Table 4:** Participant-Reported Disorder and Disease Testing Rates—Frequencies and Percentages.

	*n*	*%*
Genetic profiling for heritable disorders	166	83.0
Hip screening (OFA, FCI, PennHIP, etc.)	130	65.0
Elbow screening (OFA, FCI, BVA, etc.)	117	58.5
Eye screening (CAER, ACES, etc.)	104	52.0
Cardiac screening	59	29.5
Patellar luxation screening	36	18.0
Thyroid screening	18	9.0
Congenital deafness screening (e.g., BAER)	10	5.0
Spinal screening	6	3.0

**Table 5:** Frequencies and Percentages for Breeding Dog Selection Considerations and Exclusionary Behaviour or Temperament Traits.

	*n*	*%*
Factors/characteristics considered when selecting breeding dogs		
Temperament	196	98.0
Results from health testing (hip scoring, DNA results, etc.)	183	91.5
Structural conformation	183	91.5
Overall health (allergies, skin conditions, etc.)	173	86.5
Pedigree	156	78.0
Physical appearance (colour, coat type, etc.)	148	74.0
How much you personally like the dog	69	34.5
Performance status (titles, championships, etc.)	54	27.0
Working ability	52	26.0
Accessibility	18	9.0
Breeding dog exclusionary behaviour/temperament traits		
Person-directed aggression	186	93.0
Fearfulness	166	83.0
Anxiety	145	72.5
Dog-directed aggression	145	72.5
Obsessive compulsive tendencies (e.g., light chasing, fly snapping)	114	57.0
Animal-directed aggression (e.g., cats, chickens, rabbits)	92	46.0
Separation anxiety	88	44.0
Low trainability	67	33.5
Excessive vocalizing (e.g., barking, howling)	64	32.0
Resource guarding	60	30.0
Noise sensitivity (e.g., fireworks, thunder)	51	25.5
Excitability/hyperactivity	50	25.0
Submissive urination	46	23.0
High prey drive	43	21.5
Inappropriate urination/defecation (e.g., in the house, car, crate)	37	18.5
Inappropriate chewing/eating (e.g., rocks, clothing, canine faeces)	30	15.0
Escaping tendencies (e.g., fence climbing/jumping)	24	12.0
Inappropriate digging	12	6.0

**Table 6:** Puppies’ exposure to common socialisation stimuli and experiences during the socialisation period (5–8 weeks; with grey shading denoting the most frequent response).

	Never		Once or Twice		Three or Four Times		Once per Week		Several Times per Week		Once per Day		Several Times per Day	
	*n*	*%*	*n*	*%*	*n*	*%*	*n*	*%*	*n*	*%*	*n*	*%*	*n*	*%*
Household noises (e.g., television)	2	1.0	3	1.5	9	4.5	3	1.5	24	12.0	17	8.5	140	70.0
Outdoor surfaces (e.g., grass, gravel)	1	0.5	2	1.0	9	4.5	4	2.0	47	23.5	26	13.0	111	55.5
Indoor surfaces (e.g., tiles, floorboards)	4	2.0	11	5.5	13	6.5	6	3.0	45	22.5	20	10.0	99	49.5
Machinery (e.g., washing machine, lawn mower)	1	0.5	7	3.5	9	4.5	8	4.0	54	27.0	37	18.5	83	41.5
Sudden/loud noises (e.g., alarms, sirens)	12	6.0	28	14.0	25	12.5	16	8.0	39	19.5	28	14.0	52	26.0
Time in confinement (e.g., crate/x-pen)	24	12.0	25	12.5	23	11.5	6	3.0	35	17.5	50	25.0	36	18.0
Diverse humans (e.g., tall/short)	2	1.0	23	11.5	32	16.0	33	16.5	82	41.0	13	6.5	15	7.5
Unfamiliar foods	8	4.0	22	11.0	25	12.5	23	11.5	80	40.0	34	17.0	8	4.0
Unfamiliar toys	1	0.5	7	3.5	19	9.5	19	9.5	72	36.0	45	22.5	37	18.5
Human apparel (e.g., umbrellas, hats)	12	6.0	25	12.5	39	19.5	18	9.0	68	34.0	24	12.0	14	7.0
Environments outside rearing domain	15	7.5	28	14.0	15	7.5	11	5.5	56	28.0	41	20.5	32	16.0
Bathing/grooming (e.g., brushing)	2	1.0	27	13.5	35	17.5	58	29.0	48	24.0	24	12.0	4	2.0
Vet visit	7	3.5	132	66.0	45	22.5	13	6.5	0	0.0	0	0.0	1	0.5
Car journeys	7	3.5	62	31.0	53	26.5	40	20.0	31	15.5	4	2.0	1	0.5
Children under 10	14	7.0	45	22.5	41	20.5	32	16.0	36	18.0	7	3.5	25	12.5
Other species (excluding dogs)	41	20.5	42	21.0	24	12.0	13	6.5	33	16.5	12	6.0	35	17.5
Unfamiliar dogs	63	31.5	43	21.5	20	10.0	25	12.5	31	15.5	9	4.5	7	3.5
Alone time from humans/littermates	61	30.5	48	24.0	25	12.5	12	6.0	29	14.5	23	11.5	2	1.0
Leashed walking	57	28.5	34	17.0	25	12.5	11	5.5	46	23.0	24	12.0	2	1.0

Note. Discrepancies between the total sample size (*N* = 200) and the summed category counts/percentages reflect missing data for participants who did not answer specific questions.

**Table 7:** Spearman’s rho correlations between human-centered socialisation experiences and breeding length (years participants have bred dogs), litter frequency (estimated number of litters over the previous five years), and breeding program size (number of dogs in breeding program).

Socialisation Experiences	Breeding Length	Litter Frequency	Breeding Program Size
Diverse humans (e.g., tall/short)	−0.21 **	−0.23 ***	−0.22 **
Children under 10 years old	−0.26 ***	−0.09	−0.16 *
Various human apparel (e.g., umbrellas, hats)	−0.20 **	−0.23 ***	−0.20 **

* *p* < 0.05, ** *p* < 0.01, *** *p* < 0.001.

## Data Availability

Data collected for this study are available on request from the corresponding author, provided that approval to release the data is obtained from the La Trobe University Human Ethics Committee.

## Supplementary Materials

The following supporting information can be downloaded at: https://www.mdpi.com/article/10.3390/ani15152302/s1, Supplementary S1: Questionnaire.
