# Supplementary material for: A Quantitative Exploration of Australian Dog Breeders’ Breeding Goals, Puppy Rearing Practices and Approaches to Socialisation
Source: Animals (Basel). 2025 Aug 6;15(15):2302. doi: 10.3390/ani15152302 (PMC12345502; doi:10.3390/ani15152302)
Supplement: Supplementary file 1 [file animals-15-02302-s001.zip › animals-3768381-supplementary.pdf]

[Attachment: "Breeder Questionnaire PICF.pdf"]

Consent Form – Declaration by Participant

I (the participant) have read and understood the Participant Information Statement, and any questions have been answered to my satisfaction. I agree to participate in the study, I know I can withdraw whilst completing the questionnaire and after if I provide my email address at the end of the questionnaire. I agree information provided by me or with my permission during the project may be included in a thesis, presentation and published in journals on the condition that I cannot be identified. I understand my data are to be used in this research project and could also be used in related projects conducted by the same research group.

Do you agree to participate?

◯ I agree, start questionnaire
◯ No, I do not wish to participate

In this study you will encounter a series of questions concerning aspects of your breeding program, from breeding dog selection to potential owner identification and contact. Please answer to the best of your ability. This questionnaire should take you 20-30 minutes to complete.

Your Breeding Program

For how long have you been breeding dogs?

◯ 1 year or less
◯ 2 years
◯ 3 years
◯ 4 years
◯ 5 years
◯ 6 years
◯ 7 years
◯ 8 years
◯ 9 years
◯ 10 years
◯ 11 years
◯ 12 years
◯ 13 years
◯ 14 years
◯ 15 years
◯ 16 years
◯ 17 years
◯ 18 years
◯ 19 years
◯ 20 years or longer

Why do you choose to breed dogs? Please select all that apply.

☐ For pleasure (i.e. a hobby)
☐ To improve the breed(s) I have chosen
☐ For profit/financial gain
☐ To produce companion dogs for others (i.e. supply 'pets')
☐ To produce companion dogs for yourself
☐ To produce performance dogs for others (e.g. for breeding, showing, sporting, working, etc.)
☐ To produce performance dogs for myself (e.g. for breeding, showing, sporting, working, etc.)
☐ Other

What is/are your 'other' reason(s) for choosing to breed dogs? Please write.

What dog type(s) or breed(s) do you currently breed? Please write.

____

What are the top three reasons you chose the dog breed/type you are currently breeding? If you breed more than one breed/type of dog, please answer regarding the one you breed most often.

☐ Public popularity
☐ Profitability
☐ Tradition (e.g. parents/grandparents bred them)
☐ Breed history/origin
☐ Physical appearance/attractiveness
☐ Behaviour/temperament traits
☐ Suitability as pets
☐ Ability to fulfill performance/working roles (e.g. hunting, protection, agility, obedience)
☐ Previous experience with the breed (e.g. close friends bred them, had one as a child)
☐ Other

What is/are your 'other' reason(s) for choosing the breed/type of dog you currently breed? Please write.

Are you a member of any canine clubs or associations (e.g. kennel club, breed/type organisation)?

◯ Yes
◯ No

Which club(s)/association(s) are you a member of? Please select all that apply.

☐ National kennel club(s)/association(s) (e.g. ANKC, AKC, KC, etc.)
☐ Private breeding club(s)/association(s) (e.g. Master Dog Breeders and Associates, Australian Association of Pet Dog Breeders, etc.)
☐ Breed/breed type club(s)/association(s) (e.g. Gun Dog Club, Dalmatian club, Australian Labradoodle Association, etc.)
☐ Other ______

How many bitches/dogs do you own that are currently in your breeding program, excluding those that are retired/neutered?

◯ None
◯ 1 bitch/dog
◯ 2 bitches/dogs
◯ 3 bitches/dogs
◯ 4 bitches/dogs
◯ 5 bitches/dogs
◯ 6 bitches/dogs
◯ 7 bitches/dogs
◯ 8 bitches/dogs
◯ 9 bitches/dogs
◯ 10 bitches/dogs
◯ 11 bitches/dogs
◯ 12 bitches/dogs
◯ 13 bitches/dogs
◯ 14 bitches/dogs
◯ 15 bitches/dogs
◯ 16 bitches/dogs
◯ 17 bitches/dogs
◯ 18 bitches/dogs
◯ 19 bitches/dogs
◯ 20 or more bitches/dogs

Some breeders place their breeding bitches/dogs into 'pet/guardian homes' under 'breeders' terms', where they live with a family and return to their breeder for breeding purposes such as mating, whelping, and puppy rearing. How many bitches/dogs do you have currently placed in homes on 'breeders' terms'?

◯ None
◯ 1 bitch/dog
◯ 2 bitches/dogs
◯ 3 bitches/dogs
◯ 4 bitches/dogs
◯ 5 bitches/dogs
◯ 6 bitches/dogs
◯ 7 bitches/dogs
◯ 8 bitches/dogs
◯ 9 bitches/dogs
◯ 10 bitches/dogs
◯ 11 bitches/dogs
◯ 12 bitches/dogs
◯ 13 bitches/dogs
◯ 14 bitches/dogs
◯ 15 bitches/dogs
◯ 16 bitches/dogs
◯ 17 bitches/dogs
◯ 18 bitches/dogs
◯ 19 bitches/dogs
◯ 20 or more bitches/dogs

How many litters of puppies do you typically breed per year?

◯ None
◯ 1 litter
◯ 2 litters
◯ 3 litters
◯ 4 litters
◯ 5 litters
◯ 6 litters
◯ 7 litters
◯ 8 litters
◯ 9 litters
◯ 10 litters
◯ 11 litters
◯ 12 litters
◯ 13 litters
◯ 14 litters
◯ 15 litters
◯ 16 litters
◯ 17 litters
◯ 18 litters
◯ 19 litters
◯ 20 or more litters

Looking back, how many litters do you estimate you've produced in the last five years?

◯ 1 litter
◯ 2 litters
◯ 3 litters
◯ 4 litters
◯ 5 litters
◯ 6 litters
◯ 7 litters
◯ 8 litters
◯ 9 litters
◯ 10 litters
◯ 11 litters
◯ 12 litters
◯ 13 litters
◯ 14 litters
◯ 15 litters
◯ 16 litters
◯ 17 litters
◯ 18 litters
◯ 19 litters
◯ 20 or more litters

Where do your breeding bitches (when not close to whelping or rearing a litter) and/or dogs reside for most of the time during the day?

◯ Inside your house
◯ Outside your house, in the backyard
◯ Outside your house, in an indoor kennel setup
◯ Outside your house, in outdoor kennel setup (i.e. yards/runs)
◯ Outside your house, with another caretaker (i.e. 'guardianship' or 'breeders terms' arrangements)
◯ Other ______

Where do your breeding bitches (when not close to whelping or rearing a litter) and/or dogs reside for most of the time at night?

◯ Inside your house
◯ Outside your house, in the backyard
◯ Outside your house, in an indoor kennel setup
◯ Outside your house, in outdoor kennel setup (i.e. yards/runs)
◯ Outside your house, with another caretaker
◯ Other ______

Do you engage in any organised activities with your breeding bitches/dogs? If so, what? Please select all that apply.

☐ Conformation showing
☐ Sports (e.g. obedience, agility, tracking, etc.)
☐ Working (e.g. guard, livestock control, hunting, etc.)
☐ Volunteer (e.g. search and rescue, therapy, etc.)
☐ Other ______
☐ I don't engage in any organised activities with my breeding dogs

Where do you currently obtain your breeding bitches/dogs, or where would you expect to obtain them in the future? Please select all that apply.

☐ Breed and raise them myself
☐ Friends/associates who are breeders
☐ Breeders I don't know (e.g. classified ads, word of mouth, etc.)
☐ Other ______

Do you conduct any health testing on your bitches/dogs to evaluate their suitability for breeding?

◯ Yes
◯ No

Which of the following health tests do you typically conduct on your breeding bitches/dogs? Please select all that apply.

☐ Genetic profiling for heritable disorders (MyDogDNA, Orivet, Embark, etc.)
☐ Hip screening (OFA, FCI, BVA, PennHIP, etc.)
☐ Elbow screening (OFA, FCI, BVA, etc.)
☐ Eye screening (CAER, ACES, BVA/KC/ISDS Eye Schemes, etc.)
☐ Patellar luxation screening
☐ Cardiac screening
☐ Congenital deafness screening (e.g. BAER)
☐ Spinal screening
☐ Thyroid screening
☐ Other ______

When selecting bitches/dogs for your breeding program, which of the following characteristics do you consider? Please select all that apply.

☐ Temperament
☐ How much you personally like the dog
☐ Overall health (allergies, skin conditions, etc.)
☐ Performance status (titles, championships, etc.)
☐ Results from health testing (hip scoring, eye testing, DNA results, etc.)
☐ Physical appearance (colour, coat type, etc.)
☐ Structural conformation
☐ Working ability
☐ Pedigree
☐ Accessibility
☐ Other ______
☐ None of these

Which of the following behaviours/temperament traits, if displayed, would lead you to exclude a bitch/dog from your breeding program? Please select all that apply.

☐ Person-directed aggression
☐ Anxiety
☐ Excessive vocalising (e.g. barking, howling, whining)
☐ Excitability/hyperactivity
☐ Fearfulness
☐ Inappropriate urination/defecation (e.g. in the house, car, their crate)
☐ Dog-directed aggression
☐ Animal-directed aggression (e.g. cats, chickens, rabbits)
☐ High prey-drive
☐ Inappropriate chewing/eating (e.g. rocks, clothing, canine faeces)
☐ Inappropriate digging
☐ Escaping tendencies (e.g. fence climbing/jumping, opening doors)
☐ Separation anxiety
☐ Obsessive compulsive tendencies (e.g. obsessive self-grooming, tail chasing, light chasing, fly snapping)
☐ Low trainability
☐ Resource guarding
☐ Noise sensitivity (e.g. fireworks, thunder/storms)
☐ Submissive urination
☐ Other ______

How often do you use stud dogs from outside of your breeding program?

Never — About half the time — Always

(Place a mark on the scale above)

When using stud dogs outside of your breeding program, how often do you meet them prior to breeding?

Never — About half the time — Always

(Place a mark on the scale above)

How often do you use artificial insemination techniques utilising dogs from outside of your breeding program (e.g. frozen semen from a dog located interstate/internationally)?

Never — About half the time — Always

(Place a mark on the scale above)

How many litters do your dogs (i.e. males) typically produce prior to retirement from breeding?

◯ 1
◯ 2-3
◯ 4-5
◯ 6-10
◯ 10-20
◯ More than 20

How many litters do your bitches typically produce prior to retirement from breeding?

◯ 1
◯ 2-3
◯ 4-5
◯ 6 or more

Once your breeding dogs retire, do you typically:

◯ Retain them
◯ Rehome them with friends/family members
◯ Rehome them with a member of the public
◯ Other ______

What degree of impact do you think the COVID-19 pandemic has had on your ability to breed dogs (e.g. find suitable breeding stock, plan and prepare for litters, raise puppies, etc.)?

No impact at all | Some impact | A large impact

(Place a mark on the scale above)

What degree of impact do you think the COVID-19 pandemic has had on your ability to sell your puppies (e.g. find suitable owners, have owners visit their puppies, transporting puppies to their new home)?

No impact at all | Some impact | A large impact

(Place a mark on the scale above)

Raising Your Puppies Whelping and General Rearing

Where do your mother dogs typically whelp their puppies?

◯ Inside your house
◯ Outside your house, in the backyard
◯ Outside your house, in the garage/shed
◯ Outside your house, in a kennel setup
◯ Outside your house, with another caretaker (i.e. off-site)
◯ At a veterinary or specialised reproductive facility
◯ Other ______

How often do your bitches whelp under human supervision?

Never | About half the time | Always

(Place a mark on the scale above)

How often do your bitches whelp via vaginal delivery?

Never | About half the time | Always

(Place a mark on the scale above)

At what age do your puppies typically first experience human handling?

◯ During the whelping process or immediately after birth
◯ Within a few hours of birth
◯ Between 1-7 days of age
◯ Between 8 days and 14 days of age
◯ Between 2-3 weeks of age
◯ Not before 3 weeks of age

From when your puppies are born until their eyes/ears open, where do they spend most of their time?

◯ Inside your house
◯ Outside your house, in the backyard
◯ Outside your house, in the garage/shed
◯ Outside your house, in a kennel setup
◯ Outside your house, with another caretaker (i.e. off-site)
◯ Other ______

From when your puppies are 2 weeks of age until they leave the whelping area, where do they spend most of their time?

◯ Inside your house
◯ Outside your house, in the backyard
◯ Outside your house, in the garage/shed
◯ Outside your house, in a kennel setup
◯ Outside your house, with another caretaker (i.e. off-site)
◯ Other ______

Once you move your puppies from their whelping area (e.g. whelping box, c-crate), where do they spend most of their time?

◯ Inside your house
◯ Outside your house, in the backyard
◯ Outside your house, in the garage/shed
◯ Outside your house, in a kennel setup
◯ Outside your house, with another caretaker (i.e. off-site)
◯ Other ______

Please use the sliding scales below to indicate how important you feel it is for you to control/monitor the following environmental aspects and experiences during the first six weeks of rearing a litter of puppies.

Flooring in the whelping/rearing areas

Not at all important — Somewhat important — Very important

(Place a mark on the scale above)

Environmental temperature

Not at all important — Somewhat important — Very important

(Place a mark on the scale above)

Exposure to drafts/winds

Not at all important — Somewhat important — Very important

(Place a mark on the scale above)

Exposure to rain

Not at all important — Somewhat important — Very important

(Place a mark on the scale above)

The mother's nutrition

Not at all important — Somewhat important — Very important

(Place a mark on the scale above)

The puppies' nutrition

Not at all important — Somewhat important — Very important

(Place a mark on the scale above)

Exposure to other animals (i.e. excluding the mother)

Not at all important — Somewhat important — Very important

(Place a mark on the scale above)

Exposure to people from outside the household/property

Not at all important — Somewhat important — Very important

(Place a mark on the scale above)

How many people, including yourself, are involved in the day-to-day husbandry and upkeep of your puppies (i.e. feeding, cleaning, and handling)?

◯ 1 person
◯ 2 people
◯ 3 people
◯ 4 people
◯ 5 people
◯ 6 people
◯ 7 people
◯ 8 people
◯ 9 people
◯ 10 or more people

At what age do your puppies typically begin weaning onto solid foods?

◯ 2 weeks of age
◯ 3 weeks of age
◯ 4 weeks of age
◯ 5 weeks of age
◯ 6 weeks of age
◯ 7 weeks of age
◯ 8 weeks of age or older

How often do your puppies receive a veterinary health check prior to leaving for their new homes?

Never — About half the time — Always

(Place a mark on the scale above)

How often are your puppies microchipped prior to leaving for their new homes?

Never — About half the time — Always

(Place a mark on the scale above)

How often are your puppies typically 'up to date' with vaccinations prior to leaving for their new homes?

Never — About half the time — Always

(Place a mark on the scale above)

How often are your puppies typically 'up to date' with worming prior to leaving for their new homes?

Never | About half the time | Always

(Place a mark on the scale above)

Puppy Handling, Experiences, and Socialisation

How often are your puppies handled and/or interacted with whilst their siblings are present (e.g. picked up, stroked, played with, or handled with their littermates beside them or very close by) by humans?

◯ Several times a day
◯ Once a day
◯ Once every few days
◯ Once a week or less

How often are your puppies handled and/or interacted with one-on-one (e.g. picked up, stroked, played with, or handled to check weight/condition away from their littermates) by humans?

◯ Several times a day
◯ Once a day
◯ Once every few days
◯ Once a week or less
◯ I never interact with my puppies one-on-one away from their littermates

Do you practice any standardised or formal socialisation protocols? If so, which one(s)? Please select all that apply.

☐ No, I don't practice a standardised or formal socialisation protocol
☐ Early Neurological Stimulation
☐ Puppy Culture
☐ Avidog
☐ Other ______

How important a role do you think breeders play in the development of their puppy’s adult behaviour and temperament?

Not at all important | Somewhat important | Very important

(Place a mark on the scale above)

How impactful do you believe early experiences (i.e. before they leave for their new home) are on a dog's adult behaviour and temperament?

No impact at all | Some impact | A large impact

(Place a mark on the scale above)

How impactful do you believe later experiences (i.e. after they leave for their new home) are on a dog's adult behaviour and temperament?

No impact at all | Some impact | A large impact

(Place a mark on the scale above)

Please use the scales below to indicate how often your puppies encounter the following stimuli and/or experiences when they are 5 to 8 weeks of age (from never to several times per day).

	never	once or twice	three or four times	once a week	several times per week	once per day	several times per day
Offered unfamiliar foods (e.g. a new type of treat)	○	○	○	○	○	○	○
Experiences with a diverse range of other humans (e.g. tall/short, elderly, with disability)	○	○	○	○	○	○	○
Experiences with children under 10 years old	○	○	○	○	○	○	○
Experiences with other animal species (i.e. excluding dogs)	○	○	○	○	○	○	○
Environments outside the rearing domain	○	○	○	○	○	○	○
Experiences with unfamiliar dogs	○	○	○	○	○	○	○
Exposure to common indoor surfaces (e.g. tiles, floorboards)	○	○	○	○	○	○	○
Exposure to common outdoor surfaces (e.g. grass, gravel, concrete)	○	○	○	○	○	○	○
Experiences with leashed walking	○	○	○	○	○	○	○
Time spent in confinement (e.g. in a crate or x-pen)	○	○	○	○	○	○	○
Experiences with bathing/grooming (e.g. brushing, nail clipping)	○	○	○	○	○	○	○
Experiences with various human apparel (e.g. umbrellas, hats, large coats)	○	○	○	○	○	○	○
Experiences with car journeys	○	○	○	○	○	○	○
Exposure to household noises (e.g. telephone, television)	○	○	○	○	○	○	○
Exposure to machinery (e.g. cars, washing machines, lawnmowers)	○	○	○	○	○	○	○
Sudden and/or loud noises (e.g. alarms, sirens, fireworks)	○	○	○	○	○	○	○
Veterinary surgery visit	○	○	○	○	○	○	○
Time spent alone, away from littermates and/or familiar humans	○	○	○	○	○	○	○
Offered unfamiliar toys	○	○	○	○	○	○	○

What degree of impact do you think that the COVID-19 pandemic has had on your ability to rear/socialise your puppies?

No impact at all — Somewhat of an impact — A big impact

(Place a mark on the scale above)

Homing Your Puppies

What type of homes do your puppies primarily go to?

◯ Pet/companionship
◯ Sporting (e.g. agility, obedience, etc.)
◯ Working (e.g. assistance work, hunting, etc.)
◯ Conformation Showing
◯ Other ______

How particular (i.e. "choosy") are you when it comes to selecting owners for your puppies?

Not at all particular | Somewhat particular | Very particular

(Place a mark on the scale above)

At what age do you typically allow potential owners to reserve a puppy from a litter?

◯ Before the puppies are born
◯ As soon as or not long after the puppies are born (i.e. within the first few days)
◯ Within their first few weeks of life
◯ Between 4-7 weeks of age
◯ Not until the puppies are ready to leave

At what age do you typically 'match up' potential owners to the puppies they are receiving?

◯ Before the puppies are born
◯ As soon as or not long after the puppies are born (i.e. within the first few days)
◯ Within their first few weeks of life
◯ Between 4-7 weeks of age
◯ Not until the puppies are ready to leave

When 'matching up' owners to your puppies, which of the following factors do you take into consideration? Please select all that apply.

☐ Owner preferences (sex, colour, etc.)
☐ Owner characteristics (work schedule, previous experience, etc.)
☐ Puppy characteristics (temperament, behaviour, etc.)
☐ Owner/puppy dynamic during interactions
☐ Order of contact (i.e. 'first in best dressed')
☐ Other ______
☐ None of the above

When selling puppies, how much control do owners have over which puppy they are offered/receive?

0% control | 50% control | 100% control

(Place a mark on the scale above)

At what age do your puppies typically leave for their new home?

◯ Before 6 weeks of age
◯ 6-7 weeks of age
◯ 8-9 weeks of age
◯ 10-12 weeks of age
◯ 12-14 weeks of age
◯ After 14 weeks of age

Disregarding current legislation, what age do you think your puppies should leave for their new home?

◯ Before 6 weeks of age
◯ 6-7 weeks of age
◯ 8-9 weeks of age
◯ 10-12 weeks of age
◯ 12-14 weeks of age
◯ After 14 weeks of age

Do you allow confirmed puppy buyers to visit your premises to tour your breeding program, such as meeting your adult dogs, viewing your kennels/living arrangements, and/or meeting their puppy or other available puppies prior to collection?

◯ Yes
◯ No

What percentage of confirmed puppy buyers visit your premises to tour your breeding program prior to collecting their puppy (i.e. to tour your facilities, meet you and your dogs ect.)?

0% of owners | 50% of owners | 100% of owners

(Place a mark on the scale above)

What percentage of confirmed puppy buyers visit your premises or where the litter is located for the purposes of meeting the litter of puppies and/or their specific puppy before taking them home (e.g. prior to the puppy being old enough to leave the litter)?

0% of owners | 50% of owners | 100% of owners

(Place a mark on the scale above)

From what age do/would you allow confirmed puppy buyers to visit their puppy before collection (i.e. when the puppies are old enough to leave your care)?

◯ As soon as or not long after the puppies are born (i.e. within the first few days)
◯ Within their first few weeks of life
◯ Between 4-7 weeks of age
◯ 8 weeks of age or older
◯ Only in the few days prior to collecting their puppy
◯ I do not/would not allow puppy buyers to visit their puppy prior to collecting them

---

Do you allow potential owners (i.e. interested in but have not yet agreed to purchase a puppy) to visit your premises to tour your breeding program, such as meeting your adult dogs, viewing your kennels/living arrangements, and/or meeting any available puppies?

◯ Yes
◯ No

What percentage of potential owners visit your premises to tour your breeding program prior to deciding to purchase a puppy from you?

0% of potential owners | 50% of potential owners | 100% of potential owners

(Place a mark on the scale above)

When your puppies go to their new home, do/would you provide a ‘going home’ pack?

◯ Yes
◯ No

What do/would you include in your 'going home' pack? Please select all that apply.

☐ Reading materials (breed information, puppy rearing guidelines, etc.)
☐ Puppy paperwork (vaccination/microchip certificate, contracts, etc.)
☐ Collar/harness and leash
☐ Supply of current food
☐ Product samples (bowls, treats, etc.)
☐ Familiar object (toy, blanket, etc.)
☐ Other ______

What percentage of owners do you maintain contact with once they collect their puppy?

0% of owners | 50% of owners | 100% of owners

(Place a mark on the scale above)

How long do/would you typically maintain contact with owners for?

◯ The first few days after they receive their puppy
◯ 1-4 weeks
◯ A couple of months
◯ For the puppy's first year of life
◯ Indefinitely

Information and Regulation

When obtaining information about how best to breed dogs and raise puppies, which of the following sources of information are you likely to consult? Please select all that apply.

☐ Federal/State/local government agencies
☐ Other breeders
☐ Scientific studies and research
☐ Breed or Interest Organisations (FCI, UKC, ANKC, AAPDB, etc.)
☐ Animal Protection Societies (ASPCA, RSPCA, etc.)
☐ General internet searches (Google, Bing, etc.)
☐ Veterinarians or other animal health practitioners
☐ Pet shops or other animal retailers
☐ Social networking sites (e.g. Facebook, Twitter)
☐ Friends or family members
☐ Other ______

How regulated do you think the dog breeding industry should be?

Not at all regulated — Somewhat regulated — Very regulated

(Place a mark on the scale above)

How regulated do you think the dog breeding industry is where you are located?

Not at all regulated — Somewhat regulated — Very regulated

(Place a mark on the scale above)

Using the sliding scales below, please indicate how important a role you think the following bodies have in the guidance and regulation of dog breeding practices.

Federal/State government agencies

Not at all important — Somewhat important — Very important

(Place a mark on the scale above)

Local government (councils, shires, counties, etc.)

Not at all important — Somewhat important — Very important

(Place a mark on the scale above)

Scientists/Researchers

Not at all important — Somewhat important — Very important

(Place a mark on the scale above)

Breed or Interest Organisations (FCI, UKC, ANKC, AAPDB, etc.)

Not at all important — Somewhat important — Very important

(Place a mark on the scale above)

Animal Protection Societies (ASPCA, RSPCA, etc.)

Not at all important — Somewhat important — Very important

(Place a mark on the scale above)

Veterinarians or other animal health practitioners

Not at all important — Somewhat important — Very important

(Place a mark on the scale above)

Pet shops or other animal retailers	Not at all important — Somewhat important — Very important<br>*(Place a mark on the scale above)*
Puppy buyers	Not at all important — Somewhat important — Very important<br>*(Place a mark on the scale above)*
Dog breeders	Not at all important — Somewhat important — Very important<br>*(Place a mark on the scale above)*

Whilst the research team have done their best to cover all relevant domains, we understand that dog breeding is a many faceted process and there will be areas that we have failed to include. If there is anything else that you think is worth mentioning in relation to your breeding program, puppy rearing practices, or anything else covered in this questionnaire, please use the text box below.

All About You Lastly, we have a few questions about you.

In what year were you born?

◯ 2004
◯ 2003
◯ 2002
◯ 2001
◯ 2000
◯ 1999
◯ 1998
◯ 1997
◯ 1996
◯ 1995
◯ 1994
◯ 1993
◯ 1992
◯ 1991
◯ 1990
◯ 1989
◯ 1988
◯ 1987
◯ 1986
◯ 1985
◯ 1984
◯ 1983
◯ 1982
◯ 1981
◯ 1980
◯ 1979
◯ 1978
◯ 1977
◯ 1976
◯ 1975
◯ 1974
◯ 1973
◯ 1972
◯ 1971
◯ 1970
◯ 1969
◯ 1968
◯ 1967
◯ 1966
◯ 1965
◯ 1964
◯ 1963
◯ 1962
◯ 1961
◯ 1960
◯ 1959
◯ 1958
◯ 1957
◯ 1956
◯ 1955
◯ 1954
◯ 1953
◯ 1952
◯ 1951
◯ 1950
◯ 1949
◯ 1948
◯ 1947
◯ 1946
◯ 1945
◯ 1944
◯ 1943
◯ 1942
◯ 1941
◯ 1940
◯ 1939
◯ 1938
◯ 1937

○ 1936
○ 1935
○ 1934
○ 1933
○ 1932
○ 1931
○ 1930
○ 1929
○ 1928
○ 1927
○ 1926
○ 1925
○ 1924
○ 1923
○ 1922
○ 1921
○ 1920
○ 1919
○ 1918
○ 1917
○ 1916
○ 1915
○ 1914
○ 1913
○ 1912
○ 1911
○ 1910
○ 1909
○ 1908
○ 1907
○ 1906
○ 1905
○ 1904
○ 1903
○ 1902
○ 1901
○ 1900

Which of the following best describes your gender identity?

○ Female
○ Male
○ Non-binary/third gender
○ Prefer to self-describe ______
○ Prefer not to say

In which country do you reside?

◯ Afghanistan
◯ Åland Islands
◯ Albania
◯ Algeria
◯ American Samoa
◯ Andorra
◯ Angola
◯ Anguilla
◯ Antarctica
◯ Antigua and Barbuda
◯ Argentina
◯ Armenia
◯ Aruba
◯ Australia
◯ Austria
◯ Azerbaijan
◯ Bahamas
◯ Bahrain
◯ Bangladesh
◯ Barbados
◯ Belarus
◯ Belgium
◯ Belize
◯ Benin
◯ Bermuda
◯ Bhutan
◯ Bolivia (Plurinational State of)
◯ Sint Eustatius and Saba
◯ Bosnia and Herzegovina
◯ Botswana
◯ Bouvet Island
◯ Brazil
◯ British Indian Ocean Territory
◯ Brunei Darussalam
◯ Bulgaria
◯ Burkina Faso
◯ Burundi
◯ Cabo Verde
◯ Cambodia
◯ Cameroon
◯ Canada
◯ Cayman Islands
◯ Central African Republic
◯ Chad
◯ Chile
◯ China
◯ Christmas Island
◯ Cocos (Keeling) Islands
◯ Colombia
◯ Comoros
◯ Congo
◯ Democratic Republic of the
◯ Cook Islands
◯ Costa Rica
◯ Côte d'Ivoire
◯ Croatia
◯ Cuba
◯ Curaçao
◯ Cyprus
◯ Czechia
◯ Denmark
◯ Djibouti
◯ Dominica
◯ Dominican Republic
◯ Ecuador
◯ Egypt
◯ El Salvador
◯ Equatorial Guinea

◯ Eritrea
◯ Estonia
◯ Eswatini
◯ Ethiopia
◯ Falkland Islands (Malvinas)
◯ Faroe Islands
◯ Fiji
◯ Finland
◯ France
◯ French Guiana
◯ French Polynesia
◯ French Southern Territories
◯ Gabon
◯ Gambia
◯ Georgia
◯ Germany
◯ Ghana
◯ Gibraltar
◯ Greece
◯ Greenland
◯ Grenada
◯ Guadeloupe
◯ Guam
◯ Guatemala
◯ Guernsey
◯ Guinea
◯ Guinea-Bissau
◯ Guyana
◯ Haiti
◯ Heard Island and McDonald Islands
◯ Holy See
◯ Honduras
◯ Hong Kong
◯ Hungary
◯ Iceland
◯ India
◯ Indonesia
◯ Iran (Islamic Republic of)
◯ Iraq
◯ Ireland
◯ Isle of Man
◯ Israel
◯ Italy
◯ Jamaica
◯ Japan
◯ Jersey
◯ Jordan
◯ Kazakhstan
◯ Kenya
◯ Kiribati
◯ Korea (Democratic People's Republic of)
◯ Republic of
◯ Kuwait
◯ Kyrgyzstan
◯ Lao People's Democratic Republic
◯ Latvia
◯ Lebanon
◯ Lesotho
◯ Liberia
◯ Libya
◯ Liechtenstein
◯ Lithuania
◯ Luxembourg
◯ Macao
◯ Madagascar
◯ Malawi
◯ Malaysia
◯ Maldives
◯ Mali
◯ Malta
◯ Marshall Islands

◯ Martinique
◯ Mauritania
◯ Mauritius
◯ Mayotte
◯ Mexico
◯ Micronesia (Federated States of)
◯ Republic of
◯ Monaco
◯ Mongolia
◯ Montenegro
◯ Montserrat
◯ Morocco
◯ Mozambique
◯ Myanmar
◯ Namibia
◯ Nauru
◯ Nepal
◯ Netherlands
◯ New Caledonia
◯ New Zealand
◯ Nicaragua
◯ Niger
◯ Nigeria
◯ Niue
◯ Norfolk Island
◯ North Macedonia
◯ Northern Mariana Islands
◯ Norway
◯ Oman
◯ Pakistan
◯ Palau
◯ State of
◯ Panama
◯ Papua New Guinea
◯ Paraguay
◯ Peru
◯ Philippines
◯ Pitcairn
◯ Poland
◯ Portugal
◯ Puerto Rico
◯ Qatar
◯ Réunion
◯ Romania
◯ Russian Federation
◯ Rwanda
◯ Saint Barthélemy
◯ Saint Helena, Ascension and Tristan da Cunha
◯ Saint Kitts and Nevis
◯ Saint Lucia
◯ Saint Martin (French part)
◯ Saint Pierre and Miquelon
◯ Saint Vincent and the Grenadines
◯ Samoa
◯ San Marino
◯ Sao Tome and Principe
◯ Saudi Arabia
◯ Senegal
◯ Serbia
◯ Seychelles
◯ Sierra Leone
◯ Singapore
◯ Sint Maarten (Dutch part)
◯ Slovakia
◯ Slovenia
◯ Solomon Islands
◯ Somalia
◯ South Africa
◯ South Georgia and the South Sandwich Islands
◯ South Sudan
◯ Spain

◯ Sri Lanka
◯ Sudan
◯ Suriname
◯ Svalbard and Jan Mayen
◯ Sweden
◯ Switzerland
◯ Syrian Arab Republic
◯ Province of China
◯ Tajikistan
◯ United Republic of
◯ Thailand
◯ Timor-Leste
◯ Togo
◯ Tokelau
◯ Tonga
◯ Trinidad and Tobago
◯ Tunisia
◯ Turkey
◯ Turkmenistan
◯ Turks and Caicos Islands
◯ Tuvalu
◯ Uganda
◯ Ukraine
◯ United Arab Emirates
◯ United Kingdom of Great Britain and Northern Ireland
◯ United States of America
◯ United States Minor Outlying Islands
◯ Uruguay
◯ Uzbekistan
◯ Vanuatu
◯ Venezuela (Bolivarian Republic of)
◯ Viet Nam
◯ Virgin Islands (British)
◯ Virgin Islands (U.S.)
◯ Wallis and Futuna
◯ Western Sahara
◯ Yemen
◯ Zambia
◯ Zimbabwe

In which state do you reside?

◯ Alabama
◯ Alaska
◯ Arizona
◯ Arkansas
◯ California
◯ Colorado
◯ Connecticut
◯ Delaware
◯ Florida
◯ Georgia
◯ Hawaii
◯ Idaho
◯ Illinois
◯ Indiana
◯ Iowa
◯ Kansas
◯ Kentucky
◯ Louisiana
◯ Maine
◯ Maryland
◯ Massachusetts
◯ Michigan
◯ Minnesota
◯ Mississippi
◯ Missouri
◯ Montana
◯ Nebraska
◯ Nevada
◯ New Hampshire
◯ New Jersey
◯ New Mexico
◯ New York
◯ North Carolina
◯ North Dakota
◯ Ohio
◯ Oklahoma
◯ Oregon
◯ Pennsylvania
◯ Rhode Island
◯ South Carolina
◯ South Dakota
◯ Tennessee
◯ Texas
◯ Utah
◯ Vermont
◯ Virginia
◯ Washington
◯ West Virginia
◯ Wisconsin
◯ Wyoming

In which state/territory do you reside?

◯ Australian Capital Territory
◯ New South Wales
◯ Northern Territory
◯ Queensland
◯ South Australia
◯ Tasmania
◯ Victoria
◯ Western Australia

Which of the following best describes where you live?

◯ Urban (inner city)
◯ Suburban (over 10 kilometres/6 miles from the inner city)
◯ Regional city (population (50,000 or more)
◯ Country town/island (population less than 50,000)
◯ Rural (not in a city or a town)

What is the highest level of education you have completed?

◯ No formal schooling
◯ Year 10 or below (up to age 16 years)
◯ Year 11 or 12 (above age 16 years)
◯ Certificate, diploma, advanced diploma, associate degree, technical/trade qualification, TAFE
◯ Undergraduate University (Bachelor's degree)
◯ Postgraduate University (Masters degree/PhD)
◯ Other ______

Which of the following best describes your employment status?

◯ Employed full time (35 hours or more per week)
◯ Employed part time (up to 34 hours per week)
◯ Unemployed and not currently looking for work
◯ Unemployed and currently looking for work
◯ Engaged in home duties
◯ Retired
◯ Student
◯ Unable to work

Which of the following best describes your household income, relative to others in your community?

◯ Very below average
◯ Below average
◯ Average
◯ Above Average
◯ Very above average

How many adults, over 18 years of age, not including you, typically reside in your household?

◯ None
◯ One
◯ Two
◯ Three
◯ Four
◯ Five or more

How many children, between the ages of 10 and 18 years of age, typically reside in your household?

◯ None
◯ One
◯ Two
◯ Three
◯ Four
◯ Five or more

How many children, under the age of 10 years, typically reside in your household?

◯ None
◯ One
◯ Two
◯ Three
◯ Four
◯ Five or more

Thank you for taking the time to participate in this questionnaire. This study is a part of a larger project investigating the role early experiences and breeder practices play in the development of adult dog outcomes, such as their temperament, behaviour, and relationship with their owner.

If you would like to find out more about this project and how you can participate as a breeder, please indicate below.

Would you like to find out more about this project and how you can participate as a breeder?

◯ Yes
◯ No

Email address: ________________________________

I would like to...

☐ Find out more about this project and how I can participate as a breeder
☐ Receive a summary of the results from this study
